# A Recombinant Secondary Antibody Mimic as a Target-specific Signal Amplifier and an Antibody Immobilizer in Immunoassays

**DOI:** 10.1038/srep24159

**Published:** 2016-04-11

**Authors:** Junseon Min, Eun Kyung Song, Hansol Kim, Kyoung Taek Kim, Tae Joo Park, Sebyung Kang

**Affiliations:** 1 Department of Biological Sciences, School of Life Sciences, Ulsan National Institute of Science and Technology (UNIST), Ulsan, 689-798, Korea; 2 Department of Chemistry, Seoul National University, Seoul, 151-747, Korea

## Abstract

We construct a novel recombinant secondary antibody mimic, GST-ABD, which can bind to the Fc regions of target-bound primary antibodies and acquire multiple HRPs simultaneously. We produce it in tenth of mg quantities with a bacterial overexpression system and simple purification procedures, significantly reducing the manufacturing cost and time without the use of animals. GST-ABD is effectively conjugated with 3 HRPs per molecule on an average and selectively bind to the Fc region of primary antibodies derived from three different species (mouse, rabbit, and rat). HRP-conjugated GST-ABD (HRP-GST-ABD) is successfully used as an alternative to secondary antibodies to amplify target-specific signals in both ELISA and immunohistochemistry regardless of the target molecules and origin of primary antibodies used. GST-ABD also successfully serves as an anchoring adaptor on the surface of GSH-coated plates for immobilizing antigen-capturing antibodies in an orientation-controlled manner for sandwich-type indirect ELISA through simple molecular recognition without any complicated chemical modification.

Binding specificity and high affinity to any given target molecule are essential features for *in vitro* and/or *in vivo* diagnostics and for many other biotechnological and biomedical applications. Antibodies have been widely used as ligands for the specific detection of biomarkers *in vitro* and/or *in vivo*, the enrichment of low abundant molecules in complex samples, and the targeted delivery of therapeutics and/or diagnostics because they have an extremely high binding affinity and specificity to their target molecules and because a variety of antibodies to virtually any desired target can be readily obtained on demand^1^,^2^,^3^,^4^. An immunoassay is a biochemical method that uses antibodies to detect the presence of specific biomolecules of interest and to measure their concentrations in a complex biological sample. Most immunoassays, including enzyme-linked immunosorbent assay (ELISA) and immunohistochemistry, commonly use two different types of antibodies or immunoglobulins (Igs), those are, primary and secondary antibodies. Each primary antibody binds to its own specific target molecules with extremely high affinity and selectivity, thus ensuring the accuracy and precision of the assay. Therefore, the appropriate primary antibodies should be chosen to guarantee the successful detection and accurate quantification of target molecules in immunoassays. On the other hand, the secondary antibodies recognize and bind to the target-bound primary antibodies regardless of the target molecules. The secondary antibodies are generally conjugated with enzymes, such as horseradish peroxidases (HRP), which repeatedly convert undetectable substrates to detectable products, resulting in significant signal amplification for the detection of low abundance target molecules. Although secondary antibodies are widely used for antibody-mediated target detection and signal amplification, the mass production of secondary antibodies requires expensive animal use as well as high manufacturing costs and time compared to those of recombinant protein production with bacterial overexpression systems. However, it is possible to replace enzyme-conjugated secondary antibodies if there are simple secondary antibody mimics that can specifically bind to the Fc region of the primary antibodies and provide sites for the conjugation of signal-amplifying enzymes simultaneously. Moreover, universal secondary antibody mimics that can broadly bind to the Fc region of the primary antibodies derived from multiple species such as mouse, rabbit, and rat are particularly useful because they would eliminate the need to prepare individual primary-secondary antibody pairs. Protein A full length protein and extracellular domain of Fcγ receptors have been used as templates for developing recombinant secondary antibody mimics, because they have an antibody-binding capability.

Sandwich-type indirect ELISA system is the most popular and convenient ELISA format, which is generally comprised of four major components: antigen-capturing antibodies, which are anchored on the surface of plates; target antigens; antigen-detecting primary antibodies; and signal-amplifying secondary antibodies. One of the most challenging tasks in this format is effectively immobilizing the antigen-capturing antibodies on the surface of the ELISA plates without significantly altering the capturing capability of the immobilized antibodies. Currently, the most widely used antibody immobilization methods are chemical modifications and the physical adsorption of antigen-capturing antibodies^5^,^6^. However, chemical immobilizations often cause significant inactivation or denaturation of the antigen-capturing antibodies, impairing their binding capability, and physically adsorbed antigen-capturing antibodies are frequently detached from the plates during the multiple washing and sample application steps of ELISA, mainly owing to the weak binding of the antibodies to the plates^5^,^6^. In addition, both chemical modification and physical adsorption of antigen-capturing antibodies often result in the immobilization of the antigen-capturing antibodies in the wrong orientation, causing an alteration of the capturing efficiency of the immobilized antibodies. Therefore, there are demands for the development of more effective and simple methods and materials for the immobilization of antigen-capturing antibodies to plates, without altering their antigen-binding capability.

In this study, we constructed a novel recombinant secondary antibody mimic (GST-ABD) by genetically combining the antibody-binding domain (ABD)^7^,^8^,^9^ with glutathione-*S*-transferase (GST) using a flexible linker. GST-ABD was produced in large quantities (>10 mg/L culture) using a bacterial overexpression system and applied to various immunoassay systems, including ELISA and immunohistochemistry, as an alternative to secondary antibodies and/or an anchoring adaptor for immobilizing antigen-capturing antibodies in sandwich-type indirect ELISA (Fig. 1). Our recombinant secondary antibody mimic, GST-ABD, can be massively produced by bacterial overexpression systems and simple purification procedures, significantly reducing the manufacturing cost and time. We successfully demonstrated that the prepared GST-ABD effectively conjugates with HRPs and selectively binds to the Fc region of primary antibodies to amplify target-specific signals in both ELISA and immunohistochemistry. We also showed that GST-ABDs effectively bind to GSH-coated plates and anchor the antigen-capturing antibodies on the surface of the plates in an orientation-controlled manner through simple molecular recognition without any complicated chemical modification.

## Results and Discussion

### GST and ABD are genetically fused via extra linker residues

**Figure 1:**
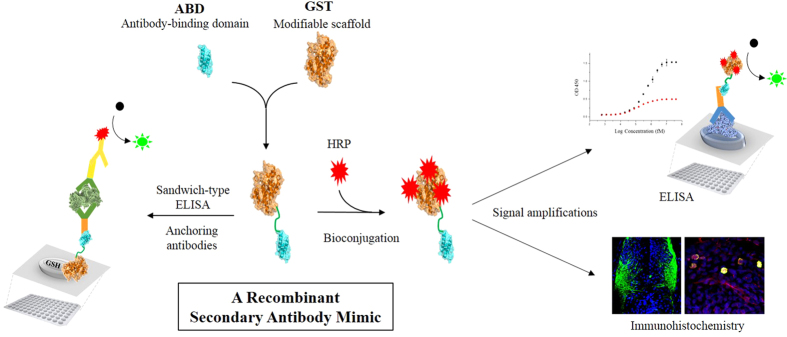
Schematic representation of the preparation of a recombinant secondary mimic, GST-ABD, and its applications to ELISA and immunohistochemistry as an alternative to secondary antibodies.

To construct a recombinant secondary antibody mimic, we prepared antibody-binding domain (ABD) isolated from protein A and genetically fused to the C-terminus of glutathione-*S*-transferase (GST) with extra linker residues (KDPNSGGGLVPRGSGGGCGGGTGGGSGGG) which offer an independency of each domain and provide an enough space for antibody access and other conjugating proteins (GST-ABD, Fig. 1). ABD of protein A, named as Z domain, has been known to specifically bind to the Fc region of immunoglobulin G (IgG) in an orientation-controlled manner without interfering antibodies’ binding capability^7^,^8^. GST is one of the most commonly used fusion tag protein that usually increases the solubility and stability of fused domains or proteins and exhibits specific interaction with glutathione (GSH) as well as provides addressable sites for additional chemical modifications, such as attachments of small chemicals and enzymes. Large amounts of GST-ABD (>10 mg/L culture) were produced in a bacterial overexpression system and were purified using simple one-step Ni-NTA agarose column chromatography, thus reducing the manufacturing cost and time significantly. High purity of GST-ABD (>99%; Figure S1A) and the successful fusion of GST and ABD with the added linker residues (Figure S1B, calc. 35890.8 Da; obs. 35890.0 Da) were confirmed with SDS-PAGE and electrospray ionization time-of-flight mass spectrometry (ESI-TOF MS), respectively. Purified GST-ABD remained intact for at least a month at 4 °C.

### GST-ABD serves as a universal IgG binding molecule, which captures free IgGs on a solid surface as well as binds to the immobilized IgGs by simple molecular recognition

**Figure 2:**
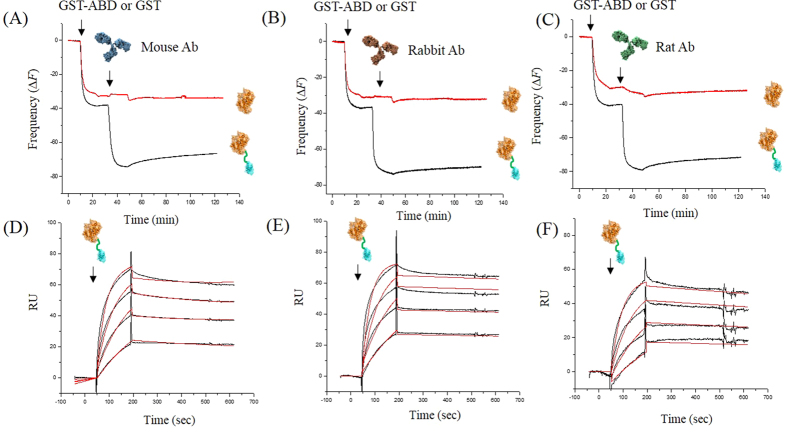
GST-ABD binds to immobilized IgGs as well as captures free IgGs derived from three different species. (**A**–**C**) QCM resonance frequency change (*ΔF*) profiles of either GST-ABD (black lines) or GST (red lines) on the gold QCM sensors and subsequent deposition of mouse (**A**), rabbit (**B**), and rat (**C**) IgGs on the monolayers of either GST-ABD (black lines) or GST (red lines). (**D**–**F**) SPR sensorgrams of GST-ABD binding to IgG-immobilized gold SPR sensors (black lines) and the fitting curves (red lines) for the mouse (**D**), rabbit (**E**), and rat (**F**) IgGs.

To investigate the binding capability of GST-ABD to antibodies derived from three different species (mouse, rabbit, and rat), we first monitored interactions between the immobilized GST-ABD and free IgGs in real-time using a quartz crystal microbalance (QCM; Fig. 2A–C)^10^,^11^. The resonance frequencies of a GST-ABD-monolayered QCM sensor drastically decreased (black lines) upon the addition of all three different types of IgGs separately, whereas those of a GST-monolayered sample (red lines) remained unchanged (Fig. 2A–C). To determine precise associating and dissociating properties of GST-ABD with various types of IgGs, we carried out surface plasmon resonance (SPR) analyses (Fig. 2D–F)^10^,^12^. In contrast to the procedure for the QCM studies, we first immobilized each IgG molecule (from mouse, rabbit, or rat) on the surfaces of standard SPR CM5 chips and then introduced GST-ABD at several different concentrations. Rapid increases and very slow decreases in the SPR response unit (RU) were observed when the solutions of GST-ABD at various concentrations and the washing buffers, respectively, were introduced (Fig. 2D–F), whereas no apparent change was observed upon introduction of GST protein and buffer (Figure S2). GST-ABD exhibited strong binding to all types of IgGs derived from three different species (Table 1). However, it bound most tightly to rabbit IgG (*K*_*d*_, 1.31 ± 0.2 nM) and least tightly to mouse IgG (*K*_*d*_, 16.8 ± 2.2 nM). The relatively fast dissociation rate constant of mouse IgGs might cause the differences in dissociation constants (Table 1). The QCM and SPR studies with various IgGs demonstrated that GST-ABD can serve as a universal IgG binding molecule, which binds to the immobilized IgGs as well as captures free IgGs on a solid surface by simple molecular recognition.

### HRP-GST-ABD is used as an effective signal-amplifying secondary antibody mimic in simple indirect ELISA

**Figure 3:**
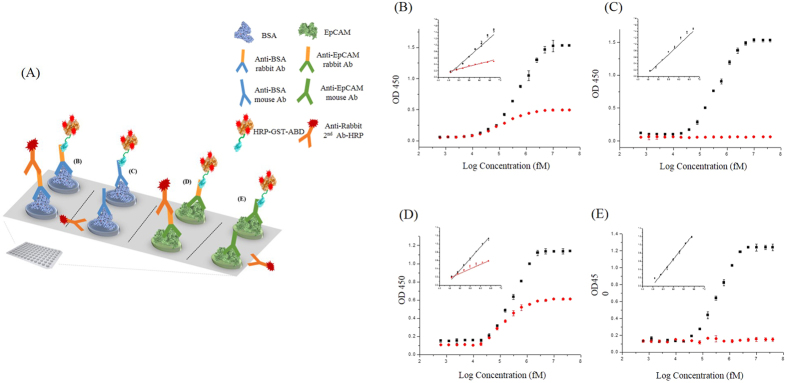
HRP-GST-ABD mimics signal-amplifying secondary antibodies in indirect ELISA. (**A**) Scheme showing different combinations of primary antibodies with either HRP-conjugated secondary antibodies or HRP-GST-ABD. (**B**–**E**) BSA (**B**,**C**) or EpCAM (**D**,**E**) are immobilized on the surface of the ELISA plates and various concentrations of either rabbit anti-BSA (**B**) or mouse anti-BSA primary antibodies (**C**) or either rabbit anti-EpCAM (**D**) or mouse anti-EpCAM primary antibodies (**E**) are applied. Linear responses of each measurement are plotted as insets of each graph.

Horseradish peroxidase (HRP) is commonly conjugated to secondary antibodies and amplifies the weak signal of a target molecule in ELISA or cell and tissue imagings^13^,^14^,^15^,^16^. Since GST-ABD has four innate cysteines in the GST portion and an additional cysteine within the linker, we attempted to conjugate the EZ-Link maleimide activated horseradish peroxidase (Mal-HRP) to GST-ABD via thiol-maleimide Michael-type addition. Consequently, we obtained HRP-conjugated GST-ABD (HRP-GST-ABD) reproducibly with three HRPs per GST-ABD molecule on average probably due to spatial constraint of conjugating sites (Figure S3). To evaluate the performance of HRP-GST-ABD as an alternative to the commonly used HRP-conjugated secondary antibodies, we first conducted a simple indirect ELISA (Fig. 3A). We immobilized a fixed amount of BSA as a model antigen on the surface of the ELISA plate via physical adsorption and applied various concentrations of rabbit anti-BSA primary antibodies. We then loaded either HRP-conjugated anti-rabbit secondary antibodies or HRP-GST-ABD after extensive washings. Subsequently, we added the o-phenylenediamine (OPD) substrate solution and measured the absorption at 450 nm immediately or 5 min later. The absorption values increased linearly in both HRP-GST-ABD and anti-rabbit HRP-labeled secondary antibody–treated samples in the range of approximately 40 pM to 5 nM, as the concentrations of primary antibody (anti-BSA IgG) increased (Fig. 3B and inset). Interestingly, HRP-GST-ABD-treated samples exhibited significantly enhanced signals compared to those seen with anti-rabbit HRP-labeled secondary antibody (Fig. 3B) at medium and high concentrations. This is possibly due to larger numbers of conjugated HRPs in the case of HRP-GST-ABD (3 HRPs per GST-ABD molecule on average, Figure S3). Although we observed significant signal enhancement at medium and high concentrations of primary antibodies, we did not see significant improvement in the limit of detection (LOD; Figure S4A, HRP-GST-ABD; 22 pM and anti-rabbit HRP-labeled secondary antibody; 25 pM). Slightly weak binding affinity of GST-ABD to the primary antibodies (*K*_d_, 1.31 nM) compared to that for primary/secondary antibody pairs (*K*_d_, hundredth of pM or lower) may limit their LOD.

Because GST-ABD can bind to not only rabbit IgG but also mouse IgG without any modification (Fig. 2), we tested whether HRP-GST-ABD can be used as a universal secondary antibody mimic against various types of IgGs derived from different species. We substituted rabbit primary anti-BSA IgG with mouse primary anti-BSA IgG and performed the same experiment as described above. While anti-rabbit HRP-labeled secondary antibody-treated samples did not show any signal, regardless of applied concentrations, HRP-GST-ABD-treated samples exhibited a graph almost identical to that of rabbit primary anti-BSA IgG (Fig. 3C). However, due to the weak binding affinity of HRP-GST-ABD to mouse primary antibodies, it took 5 min to obtain a similar LOD (29 pM) and signal response traces (Fig. 3C and S5). In addition, we performed similar experiments with the other antigenic protein, epithelial cell adhesion molecule (EpCAM), which is known as a biomarker for circulating tumor cells (CTCs)^17^,^18^. We obtained an almost identical LOD (25 pM) and linear ranges (40 pM–2.5 nM) as compared to those of BSA (Fig. 3D and S4B), when we applied rabbit primary anti-EpCAM IgG and HRP-GST-ABD. Mouse primary anti-EpCAM also exhibited almost identical results to those of BSA (Fig. 3E and S5). Together, these data suggest that HRP-GST-ABD can be applied as a signal-amplifying secondary antibody mimic for any type of antigenic molecules once corresponding primary antibodies are available, regardless of species origin (rabbit, mouse, and rat).

### GST-ABD serves as an anchoring adaptor for immobilizing antigen-capturing antibodies in sandwich-type indirect ELISA

**Figure 4:**
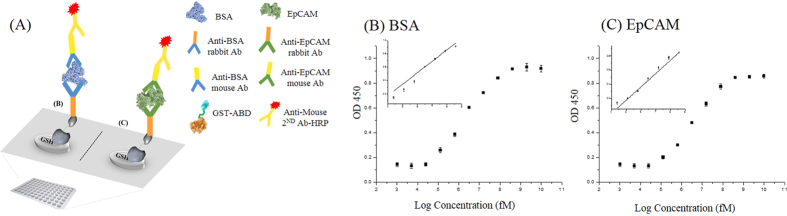
GST-ABD serves as an anchoring adaptor on the surface of GSH-coated plates for immobilizing antigen-capturing anti-bodies in an orientation-controlled manner in sandwich-type indirect ELISA. (**A**) Scheme showing the adaptation of GST-ABD as an anchoring adaptor for antigen-capturing antibodies. (**B**,**C**) GST-ABDs are spread on the surface of GSH-coated plates and saturated with capturing antibodies, either anti-BSA rabbit IgGs (**B**) or anti-EpCAM rabbit IgGs (**C**). Various amounts of BSA (**B**) or EpCAM (**C**) are added. Linear responses for each measurement are plotted as insets of each graph.

Sandwich-type indirect ELISA system is one of the most used formats. In order to capture target antigenic molecules in a complex biological sample, antigen-capturing antibodies should be securely immobilized in the proper orientation on the surface of the ELISA plates. Chemical immobilizations or related methods of immobilizing antigen-capturing antibodies often impair the antigen-binding capability of capturing antibodies due to alterations in their antigen-binding sites, denaturation, or random orientation of the antibodies^5^,^6^. Since GST-ABD has species-independent antibody-binding capability at one end (ABD) and glutathione (GSH)-binding capability at the other end (GST), the GST portion of GST-ABD will bind to the surface of the GSH-coated plates and the remaining ABD portion can bind to the Fc regions of the capturing antibodies with the proper orientation to detect their target antigens (Fig. 4A). We obtained commercially available GSH-coated plates and treated them with various concentrations of GST-ABDs. We observed the global curve, which increased as the concentration of GST-ABD increased and was finally saturated, whereas the controls (mono-streptavidin-fused ABD) did not (Figure S6). Subsequently, we determined the optimal amounts of reagents at each step in the sandwich-type indirect ELISA prior to applying this system to detecting antigens (Figures S7 and S8). We immobilized fixed amounts of GST-ABD on the surface of the GSH-coated plates, saturated them with capturing antibodies for either anti-BSA or anti-EpCAM rabbit IgGs individually, and added different amounts of BSA or EpCAM and removed unbound BSA or EpCAM with extensive washing; subsequently, we determined the amounts of antigens as done typically for a sandwich-type indirect ELISA (Fig. 4B,C). LODs (Figure S4C; BSA: 33 pM and EpCAM: 36 pM) comparable to and linear responses (range of 35 pM to 80 nM) almost identical to those of previous simple indirect ELISAs were obtained (Fig. 4). All the immobilization processes for antibodies and antigens used in this approach were achieved simply by biomolecular recognition (GST to GSH and ABD to Fc of IgGs) in an orientation-controlled manner without any additional chemical modifications. The density of antigen-capturing antibodies can be easily controlled by the amounts of anchored GST-ABD we introduced, because GST/GSH and ABD/IgG interactions are strong and specific enough. We believe our approach could minimize deviations resulted from chemical immobilizations of antigen-capturing antibodies, such as antibody inactivation or random orientation.

### HRP-GST-ABDs are as effective as HRP-conjugated secondary antibodies in cell and tissue TSA assays

**Figure 5:**
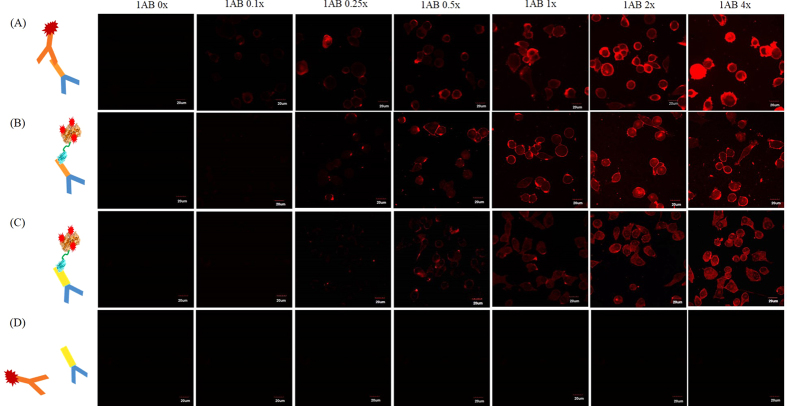
Tyramide signal amplification (TSA) assays with HRP-GST-ABD or HRP-conjugated secondary antibodies in the presence of primary antibodies derived from rabbits. SKBR3 and KB cells overexpress HER2 and integrin αβγ_3_ receptors, respectively, on their surfaces. SKBR3 (**A**,**B**) and KB (**C**,**D**) cells were treated with anti-HER2 rabbit (**A**,**B**) and anti-integrin αβγ_3_ mouse (**C**,**D**) primary IgGs, respectively, and subsequently incubated with HRP-conjugated anti-rabbit secondary antibodies (**A**,**D**) or HRP-GST-ABD (**B**,**C**) in the presence of TSA reagents. Treatment amounts for primary anti-HER2 rabbit and anti-integrin αβγ_3_ mouse antibodies are indicated at the top of each column. 1AB 1x represents the typical amount of primary antibodies used for TSA assays.

The tyramide signal amplification (TSA) assay is frequently used to detect low-abundance biomolecules such as proteins, DNA, and RNA in cells and tissues^13^,^16^. We next examined if HRP-GST-ABD can serve as a substitute for HRP-conjugated secondary antibodies in immunohistochemistry. We first performed TSA-based immunohistochemistry for two cancer cell lines, SKBR3 and KB, that overexpress HER2 and integrin αβγ_3_ receptors, respectively, on the cell surface, by using the corresponding primary IgGs (anti-HER2 rabbit and anti-integrin αβγ_3_ mouse IgGs, respectively). We first treated SKBR3 cells with anti-HER2 rabbit primary IgGs or KB cells with anti-integrin αβγ_3_ mouse IgGs and subsequently incubated them with either HRP-conjugated anti-rabbit secondary antibody or HRP-GST-ABD (Fig. 5). We observed slightly weak signal enhancements in HRP-GST-ABD treated SKBR3 and KB cells in comparison to those of HRP-conjugated anti-rabbit secondary antibody-treated SKBR3 (Fig. 5A–C) probably due to slightly weak binding affinity of GST-ABD to the primary antibodies compared to that for primary/secondary antibody pairs as we discussed in the ELISA section. Nevertheless, HRP-GST-ABD selectively bound to the surface target-bound primary antibodies and sufficiently enhanced fluorescence signal under the typical TSA condition (Fig. 5, 1AB 1x) regardless of the species origin of primary antibodies. However, we did not detect any apparent signal enhancement in KB cells treated with anti-integrin αβγ_3_ mouse IgGs and subsequently incubated with HRP-conjugated anti-rabbit secondary antibody (Fig. 5D) due to species mismatch between primary and secondary antibodies (mouse vs. rabbit). When we applied HRP-conjugated anti-mouse secondary antibody instead of anti-rabbit with anti-integrin αβγ_3_ mouse IgGs, we observed almost identical signal enhancements in both HRP-GST-ABD and HRP-conjugated secondary antibody-treated KB cells in all measured ranges (Figure S9). These results suggest again that HRP-GST-ABD can be utilized as a universal signal-amplifying secondary antibody mimic for target-cell detection once corresponding primary antibodies are available, regardless of species origin (rabbit, mouse, and rat).

**Figure 6:**
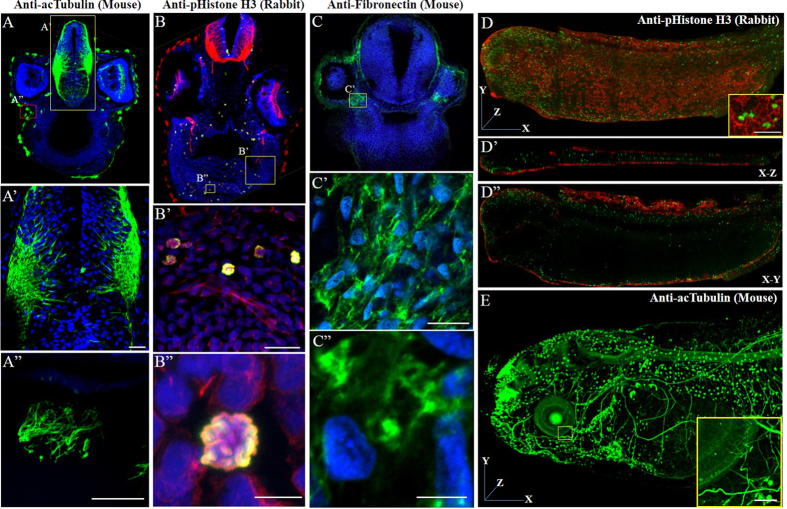
HRP-GST-ABD can effectively and efficiently amplify the signal in TSA-based immunostaining. (**A**) Anti-acetylated tubulin primary antibodies (mouse) are used to detect nerve fibers in sectioned Xenopus samples. The forebrain region is sectioned transversely and the nerve fibers are visualized with HRP-GST-ABD and tyramide-Alexa 488 (green). The nuclei are stained with DAPI in blue. The nerve fibers in the forebrain are clearly stained in green (**A’**) and the ciliary axonemes are specifically stained only in multiciliated cells (**A”**). The scale bars in a’ and a” are 20 μm and 5 μm, respectively. (**B**) Anti-phospo-histone H3 primary antibodies (rabbit) are used to detect proliferating cells in green. The tubulins are stained in red with anti-tubulin antibodies (rabbit) and the Alexa-555 conjugated anti-rabbit secondary antibodies as a counterstain after the TSA reaction. Note that the Alexa-555 conjugated anti-rabbit secondary antibodies also detected the anti-phospho-histone H3 primary anti-bodies, which are used for the TSA reaction, and the green signals amplified by HRP-GST-ABD precisely overlapped with the red signals only in the nuclei (**B’**,**B”**). The scale bars in B’ and B” are 20 μm and 5 μm, respectively. (**C**) An anti-fibronectin primary antibody (mouse) was used to detect the extracellular matrix. The scale bars in (**C**’,**C”**) are 20 μm and 5 μm, respectively. (**D**,**E**) The anti-phospho-histone H3 primary antibody (rabbit, D) or anti-acetylated tubulin primary antibody (mouse, e) are used again to stain whole mount embryos. An anti-actin antibody is used to visualize the cell boundaries in red (**D**). (**D**’,**D**”) show the confocal images in indicated sectioning planes. (**E**) HRP-GST-ABD successfully penetrates through whole mount embryo body and specifically amplified the signals of target molecules in whole mount samples. Acetylated-tubulin antibodies also detected multiciliated cells in the epidermis in addition to the nerve fibers. The scale bars in d and e are 20 μm. All control images obtained by using the regular HRP-conjugated secondary antibodies (rabbit and mouse) are presented in the supporting Figures (S10–12).

TSA-based immunohistochemistry for sectioned tissues and whole mount samples is more challenging than that of cultured cells owing to the heterogeneity and complexity of the samples as well as the requirement for higher specificity and signal-to-noise ratio^19^,^20^. To this end, we prepared *Xenopus* embryos and performed TSA-based immunostaining with HRP-GST-ABD. Stage 43 *Xenopus* embryos develop major organs and tissues and are suitable for staining of specific tissues in sectioned or whole mount samples^19^,^20^,^21^. We tested several tissue-specific antibodies, such as an anti-acetylated tubulin antibody (Fig. 6A,E and Figure S10A) and anti-myosin heavy chain (MHC) antibody (Figure S11C) to visualize the nerve fibers and muscles, respectively. We also tested anti-phospho-histone H3 (Fig. 6B,D, Figures S10C and S12A) and anti-fibronectin antibodies (Fig. 6C and Figure S11A) to detect subcellular localization of the targets in sectioned and whole mount samples. In all cases, HRP-GST-ABD resulted in high target-specific signal amplification for both sectioned (Fig. 6A–C and S11C) and whole mount samples (Fig. 6D,E) that were comparable to those of HRP-conjugated secondary antibodies (Figures S10–12) regardless of the origin of the primary antibodies (rabbit or mouse) used. Although HRP-GST-ABD exhibited slightly weaker affinity to primary antibodies in comparison to general HRP-conjugated secondary antibodies, small-sized HRP-GST-ABD may efficiently penetrate the sectioned samples and selectively bind to the target-bound primary antibodies resulting in high target-specific signal amplification. Alternatively, we could substantially reduce the sample preparation time by association with the primary antibody and HRP-GST-ABD together prior to sample treatment, in contrast to the classic sequential treatment with a primary antibody followed by that with a secondary antibody, which is necessary to avoid antibody aggregation caused by the multivalent antigen-binding sites of secondary antibodies.

Antibodies are frequently used to detect specific target molecules such as proteins and chemically labeled probes in mixed biological samples. In most cases of immuno-detection protocols, enzyme-conjugated secondary antibodies amplify signals. Although the secondary antibodies possesses very high specificity and binding affinity to primary antibodies, several limitations still need to be improved.

**Figure 7:**
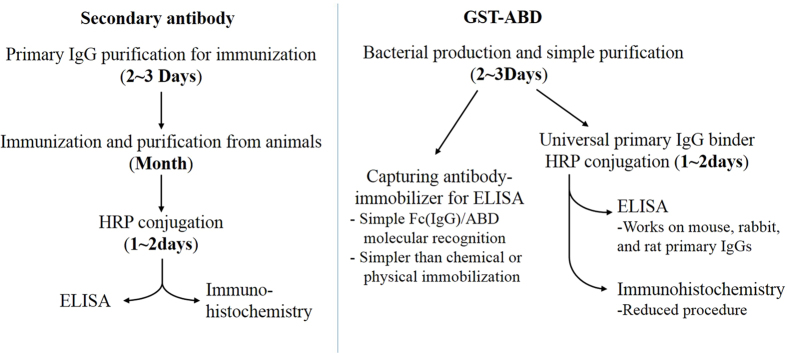
Schematic diagram comparing secondary antibodies and GST-ABD. GST-ABD can be produced with much simpler procedures without the use of animals and possesses several advantages, such as species-independency and monovalency, compared to the conventional secondary antibodies.

In this research, we have developed a novel recombinant secondary antibody mimic, GST-ABD, and produced tenth of mg quantities inexpensively using bacterial overexpression systems with a one-step purification method (Fig. 7). GST-ABD was effectively conjugated with 3 HRPs per molecule on an average and selectively bound to the Fc region of primary antibodies derived from three different species (mouse, rabbit, and rat) to amplify target-specific signals in both ELISA and immunohistochemistry (Fig. 7). GST-ABD also successfully served as an anchoring adaptor on the surface of GSH-coated plates for immobilizing antigen-capturing antibodies in an orientation-controlled manner for sandwich-type indirect ELISA. This was achieved through simple molecular interaction without any complicated chemical modifications (Fig. 7).

In additions to its versatility, GST-ABD possesses other beneficial reasons to replace secondary antibodies. All secondary antibodies are produced in higher vertebrate animals such as goat, sheep and donkey. In contrast, GST-ABD is produced in *E. coli* with much simpler procedures thereby it would minimize the use of animals and significantly reduce the batch to batch variations in its binding affinity and specificity (Fig. 7). Our approach described here may provide new opportunities to develop a simple and inexpensive immunoassay platform.

## Materials and Methods

### Construction, expression, and purification of a recombinant secondary antibody mimic (GST-ABD)

The optimized gene that encodes antibody-binding domain (ABD, 58 amino acids) with 27 extra amino acids at the N-terminus was synthesized and genetically added to the C-terminal end of glutathione-*S*-transferase (GST) containing a hexahistidine tag at the N-terminal end within the IPTG-inducible pETDuet expression vector (Invitrogen). The amplified DNAs were transformed into the competent *E. coli* strain BL21 (DE), and the recombinant secondary antibody mimic (GST-ABD) was overexpressed overnight at 30 °C. The cells were harvested by high-speed centrifugation, treated with lysozyme (50 μg/ml) at room temperature for 1 hr, sonicated for 10 min in 30-sec intervals, and subsequently centrifuged at 12,000 g for 1 hr to obtain the supernatant containing GST-ABD. The resultant GST-ABD was further purified by immobilized metal affinity chromatography (IMAC). The purity and molecular mass of GST-ABD were investigated with SDS-PAGE, UV/visible spectroscopy, and mass spectrometry.

### Quartz crystal microbalance (QCM) measurements

Standard gold QCM sensors and Q-Sense E4 (Biolin Scientific) were used as previously described, with slight modifications^10^,^11^. The overall system was operated in the flow mode with a pump maintaining temperature at 25.0 ± 0.1 °C. Each sample solution was introduced into the measurement chamber with a pump, and phosphate buffer (50 mM phosphate, 100 mM NaCl, pH 6.5) was used for washing prior to each sample application. Then, 0.1 mg/ml of GST-ABD and GST or rabbit, mouse, or rat IgGs was introduced in the same phosphate buffer. Resonance frequencies at seven harmonics (5, 15, 25, 35, 45, 55, and 65 MHz) were measured simultaneously and, for clarity, only the normalized frequencies of the third overtone have been presented^10^,^11^.

### Surface plasmon resonance (SPR) analyses

SPR experiments were conducted with carboxyl dextran CM5 standard gold sensor chips on a Biacore 3000 device at 25 °C using both a HBS-EP buffer as an immobilization solution and a PBS buffer as a running solution^10^. Rabbit, mouse, and rat IgGs were initially coupled to the carboxyl functional groups exposed on the surface of a CM5 sensor chip via standard amine-coupling chemistry. In brief, a mixture of EDC (0.5 mg/ml) and NHS (0.5 mg/ml) was gently mixed and injected onto the sensor chip at a flow rate of 10 μl/min to activate carboxyl groups of the sensor chips. Subsequently, 0.1 mg/ml of rabbit, mouse, and rat IgGs was individually injected into three different channels at the same flow rate for 7 min to immobilize them on the surfaces of the sensor chips. Unused excess reactive groups of CM5 sensor chips were blocked with 1 M ethanolamine (pH 8.0). Various amounts of GST-ABD or GST (54, 108, 216, and 432 nM) were loaded onto the IgG-immobilized CM5 sensor chips at a flow rate of 10 μl/min for 7 min. The binding and releasing kinetics of GST-ABD to various IgGs were analyzed by Biaevaluation software using the 1:1 Langmuir binding model^6^.

### Chemical conjugations of fluorescent dyes or HRP to GST-ABD

To conjugate the thiol-reactive fluorescent dye fluorescein-5-maleimide (F5M) to GST-ABD, GST-ABD was incubated with 10 mol equivalents of F5M at room temperature with vigorous shaking overnight^22^. Unreacted free F5M was removed by extensive dialysis against phosphate buffer (50 mM phosphate, 100 mM NaCl, pH 6.5) overnight. The degree of F5M conjugation to GST-ABD was evaluated with both ESI-TOF mass spectrometer and UV/Vis spectrophotometer. On an average, three fluoresceins were attached to each GST-ABD. HRP-conjugated GST-ABD (HRP-GST-ABD) was produced by conjugating EZ-Link Maleimide Activated Horseradish Peroxidase (Mal-HRP, Thermo Scientific) to GST-ABD. GST-ABD was incubated with either 2.5, 5, or 10 mol equivalents of Mal-HRP at room temperature with vigorous shaking overnight. Unconjugated free Mal-HRPs and GST-ABDs were removed by using a Centricon ultrafiltration unit (Millipore, MWCO: 100,000 Da). Treatment of GST-ABD with 10 mol equivalents of Mal-HRP resulted in complete consumption of GST-ABD to produce HRP-GST-ABD reproducibly, leaving some extra free Mal-HRPs, which were easily removed with a Centricon ultrafiltration unit.

### Simple indirect ELISA

Model antigens, BSA and EpCAM (Santa Cruz and Sino Biological Inc., respectively) were dissolved in PBS to prepare the appropriate protein solution (final concentration, 2 nM). Then, each protein solution (100 μL each) was loaded in the wells of an Immuno MicroWell 96-well plate (Maxisorp, flat-bottom, pinchibar, 400 μL) and incubated at 4 °C overnight to immobilize the target proteins on the surface of the plate through physical adsorption. The next day, each well was washed three times with 250 μL of PBS buffer with 0.1% Tween 20 (PBST). The blocking buffer (PBS, 0.5% Tween 20, 5% goat serum) was loaded (200 μL each) and incubated at 37 °C for 1 hr. After blocking, the solutions of primary antibodies against BSA (rabbit antibodies purchased from Santa Cruz Biotech and mouse antibodies from Abcam) or EpCAM (rabbit and mouse antibodies purchased from Sino Biological Inc.) were prepared with the PBS buffer by using serial dilutions. The solutions (100 μL each) were loaded in the wells of the BSA or EpCAM-immobilized plates and incubated at 37 °C for 1 hr. Each well was washed three times with 250 μL of PBST. Subsequently, 50 nM of HRP-conjugated anti-rabbit or anti-mouse secondary antibodies or HRP-GST-ABD were added (100 μL each) to the prepared sample wells and mixtures were incubated at 37 °C for 1 hr and then washed three times with 250 μL of PBST. The substrate solution of HRP was prepared using the SIGMAFAST OPD kit (Sigma-Aldrich) as per manufacturer instructions (0.4 mg/mL o-phenylenediamine [OPD], 0.4 mg/mL urea hydrogen peroxide, 0.05 M phosphate-citrate, pH 5.0) and 100 μL of the solution was loaded into each well. Reaction mixtures were incubated at room temperature for 1 or 5 min depending on the sample sets and 100 μL of 2 N H_2_SO_4_ solution was added immediately into each well to stop the reaction. The absorbance at 450 nm of each well was monitored by a multimode microplate reader, Infinite® 200 PRO (TECAN). All the experiments were conducted three times independently and plotted with errors.

### Sandwich-type indirect ELISA

GST-ABD was prepared in PBS to obtain a final concentration of 4 nM. The prepared solutions of GST-ABD (100 μL each) were loaded into the wells of a glutathione (GSH)-coated plate (clear, 8-well strips, Thermo Scientific Pierce) and incubated at 37 °C for 2 hr to allow GST-ABD to bind to immobilized GSH on the surface of plates. Each well was washed three times with 250 μL of PBST to remove excess GST-ABD. The solutions of capturing primary antibodies against BSA (rabbit IgG) and EpCAM (rabbit IgG) were prepared and added (100 μL each) to the GST-ABD-anchored wells. After the 2-hr incubation at 4 °C, each well was washed three times with 250 μL of PBST. The BSA and EpCAM solutions were prepared at various concentrations and loaded (100 μL each) independently into the prepared wells. The mixtures were incubated at 37 °C for 1 hr and each well was washed again. The solutions of detecting-primary antibodies against BSA (mouse IgG) and EpCAM (mouse IgG) were prepared and loaded (100 μL each) into the prepared wells. The mixtures were incubated at 37 °C for 1 hr and each well was washed three times with 250 μL of PBST. HRP-conjugated anti-mouse secondary antibodies (Abcam) were added (100 μL each) and incubated at 37 °C for 1 more hour, and the mixtures were washed three times with 250 μL PBST. Subsequently, 100 μL of the substrate solution was loaded into each well and the reactions were stopped 2 min later by adding 100 μL of 2 N H_2_SO_4_ solution into each well.

### TSA-based cell immunohistochemistry

Tyramide signal amplification (TSA) kits were purchased from Life Technology and all reagents were prepared per the manufacturer’s guidelines. The cell lines, SKBR3 and KB, were obtained from the Korean cell line bank (KCLB). The SKBR3 and KB cells were cultured in RPMI1640 medium with L-glutamine (300 mg/L), 10% fetal bovine serum (FBS, Invitrogen), antibiotics (1,000 units/ml penicillin, 10,000 μg/ml streptomycin, and 25 μg/ml amphotericin B), 25 mM HEPES, and 25 mM NaHCO_3_ at 37 °C under 5% CO_2_.

For the TSA assays, SKBR3 and KB cells were grown in 12-well microscopy chambers (1 × 105 cells/well), washed with PBST, fixed with 4% paraformaldehyde at 4 °C for 20 min, and finally washed again with PBST. The fixed cells were permeabilized with 0.1% Triton® X-100 solution at room temperature for 10 min and rinsed with PBST. The fixed SKBR3 and KB cells were treated with anti-HER2 rabbit primary antibodies and anti-integrin αβγ_3_ mouse primary antibodies, respectively, at room temperature for 1 hr and washed with PBST to remove unbound or non-specifically bound antibodies. The HRP-conjugated rabbit or mouse secondary antibodies were prepared by diluting the stock solution 1:100 in 1% blocking solution, and HRP-GST-ABD was prepared at equivalent amounts in the same solution. The primary antibody-treated cells were treated with the solutions of HRP-conjugated secondary antibodies or HRP-GST-ABD (100 μL each) at room temperature for 1 hr and rinsed again with PBST to remove HRP-conjugates. Tyramide working solution was freshly prepared by diluting the tyramide stock solution 1:100 in amplification buffer with 0.0015% H_2_O_2_ just before the reactions, and 100 μL was added to each sample. The reaction mixtures were incubated at room temperature for 10 min and rinsed with PBST. The nuclei of the cells were also stained with 6-diamidino-2-phenylindole (DAPI, Sigma). Fluorescence cell images were obtained using an Olympus Fluoview FV1000 confocal microscope (Olympus, UOBC).

### Embryonic sample preparation

To analyze a recombinant secondary antibody mimic (HRP-GST-ABD), we fixed embryos between stage 40 or 45 using MEMFA (MEM salts and 4% formaldehyde) or Dent’s fixative solution (methanol and 20% DMSO). Then, the fixed embryos were either used for immunostaining directly or transverse sections were prepared with 50-μm thickness at the forebrain region using a vibratome. All animal studies were performed in compliance with the guidelines of the local ethics committee for animal care and use, and were approved by the Institutional Animal Care and Use Committee of Ulsan National Institute of Science and Technology.

### TSA-based immunohistochemistry of sectioned embryonic slices and whole embryos

Embryos or sectioned embryonic slices were incubated in blocking solution (5% BSA + 2% DMSO in TBS + 0.1% Triton X-100) at room temperature for 30 min to block nonspecific binding. TSA-staining was performed with the following primary antibodies: anti-acetylated tubulin (Sigma-Aldrich), anti-phospho-histone H3 (Abcam), anti-fibronectin (DSHB), and anti-MHC (DSHB) at a 1:300 or 1:1,000 dilution for 3 hr at room temperature. The samples were rinsed with TBST (TBS + 0.1% Triton X-100). Labeling was performed with HRP-conjugated secondary antibodies (Sigma-Aldrich) or HRP-GST-ABD at 1:1,000 dilution for 2 hr. For the HRP-GST-ABD, the indicated primary antibody was pre-incubated with HRP-GST-ABD in a 1:1 ratio (0.8 ng/μl each) and the tissue samples were incubated with the mixture. Tyramide labeling was performed using tyramide Alexa-488 in amplification buffer with 0.00015% hydrogen peroxide. The mixtures were incubated at room temperature for 2 min and the tissue samples were rinsed with TBST. The nuclei were stained with DAPI (Abcam). All sectioned slices were run through washes in 100% methanol before clearing in BA:BB (benzyl alcohol/benzyl benzoate, 1:2) and mounting on slides for imaging. Images were captured using a confocal microscope (LSM700).

## Additional Information

**How to cite this article**: Min, J. *et al.* A Recombinant Secondary Antibody Mimic as a Target-specific Signal Amplifier and an Antibody Immobilizer in Immunoassays. *Sci. Rep.* **6**, 24159; doi: 10.1038/srep24159 (2016).

## Supplementary Material

Supplementary Information

## Figures and Tables

**Table 1:** Binding parameters for the interactions of GST-ABD and various types of IgGs derived from mouse, rabbit, and rat determined by SPR analyses.

IgGs	*k*_*a*_(M^−1^s^−1^)	*k*_*d*_(s^−1^)	*K*_*a*_(M^−1^)	*K*_*d*_(M)	Chi^2^
Mouse IgG	63.8 × 10^3^	1.07 × 10^**−**3^	0.59 × 10^8^	16.8 × 10^**−**9^	1.63
Rabbit IgG	66.6 × 10^3^	0.09 × 10^**−**3^	0.76 × 10^9^	1.31 × 10^**−**9^	1.32
Rat IgG	51.8 × 10^3^	0.22 × 10^**−**3^	0.23 × 10^9^	4.34 × 10^**−**9^	1.64

(*k*_a_; associating rate constant, *k*_d_; dissociating rate constant, *K*_a_; associating constant, *K*_d_; dissociating constant).
