# Supplementary material for: A Recombinant Secondary Antibody Mimic as a Target-specific Signal Amplifier and an Antibody Immobilizer in Immunoassays
Source: Sci Rep. 2016 Apr 11;6:24159. doi: 10.1038/srep24159 (PMC4827060; doi:10.1038/srep24159)
Supplement: Supplementary Information [file srep24159-s1.pdf]

Supporting information for

A Recombinant Secondary Antibody Mimic as a Target-specific Signal Amplifier and an Antibody Immobilizer in Immunoassays

Junseon Min,[1,‡,3] Eun Kyung Song,[1,‡] Hansol Kim,[1] Kyoung Taek Kim,[2] Tae Joo Park,[1,*] Sebyung Kang[1,*]

[1]Department of Biological Sciences, School of Life Sciences, Ulsan National Institute of Science and Technology (UNIST), Ulsan, 689-798, Korea and [2]Department of Chemistry, Seoul National University, Seoul, 151-747, Korea

[3]Current address: Department of Biomedical Engineering, Duke University, Durham, NC

Supporting Figures

Supporting Figure 1

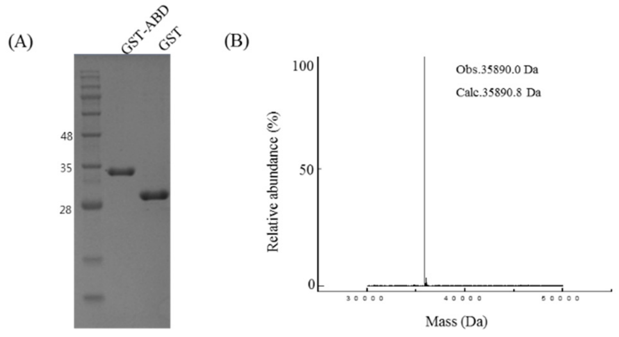

Figure S1. (A) SDS-PAGE analysis of purified GST-ABD and GST. (B) Molecular mass measurements of GST-ABD using ESI-MS. Calculated and observed masses are indicated.

Supporting Figure 2

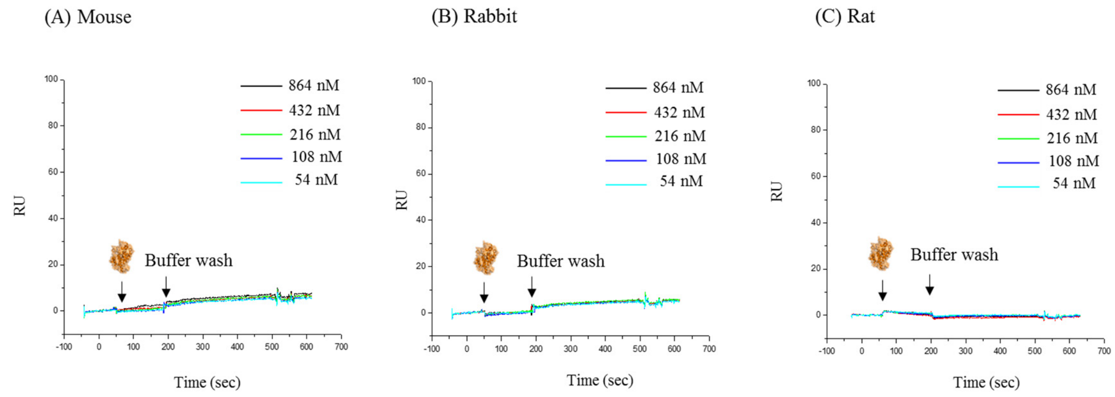

Figure S2. SPR analyses of GST binding to mouse (A), rabbit (B), and rat (C) IgG immobilized on gold SPR sensors. The time points at which GST and buffer washings are performed are indicated.

Supporting Figure 3

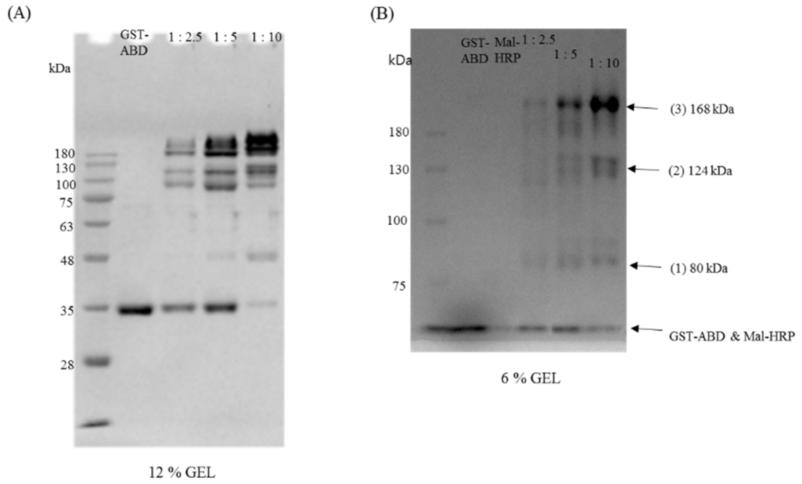

Figure S3. SDS-PAGE analysis of the reaction products obtained for GST-ABD and the EZ-Link maleimide-activated horseradish peroxidase (Mal-HRP) with various initial mixing ratios (1:0, 1:2.5, 1:5, and 1:10) as indicated. Twelve percent (A) and six percent (B) acrylamide gels with same sample sets.

Supporting Figure 4

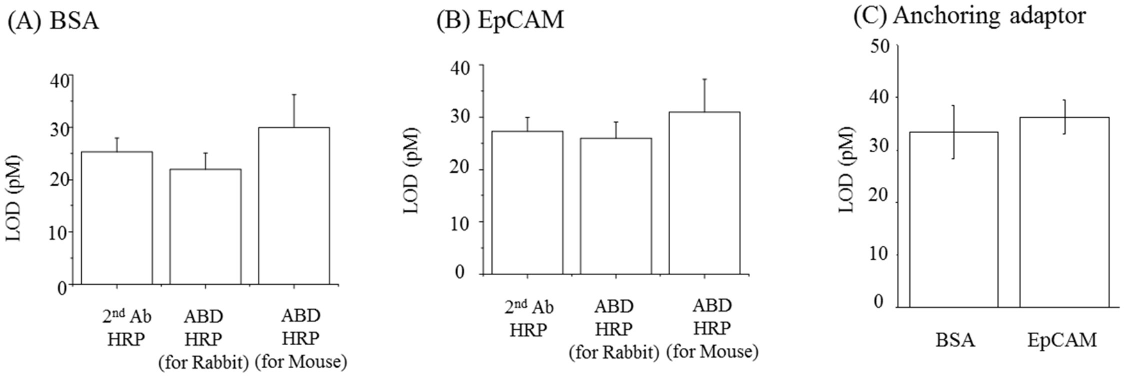

Figure S4. LODs in indirect ELISA of each measurement of BSA (A) and EpCAM (B) are plotted. LODs in sandwich-type indirect ELISA of captured BSA and EpCAM are plotted.

Supporting Figure 5

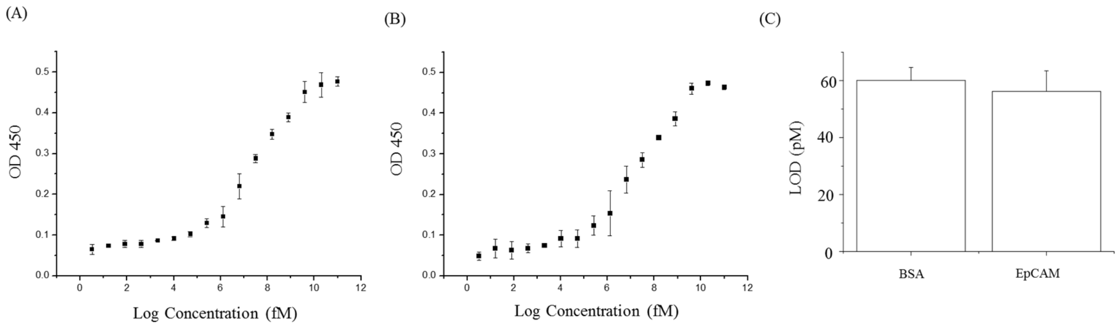

Figure S5. HRP-GST-ABD mimics signal-amplifying secondary antibodies in indirect ELISA. BSA (A) or EpCAM (B) are immobilized on the surface of the ELISA plates and various concentrations of mouse anti-BSA primary antibodies (A) or mouse anti-EpCAM primary antibodies (B) are applied. HRP-GST-ABDs are subsequently added with OPD and H_2O_2, and absorbance at 450 nm is measured immediately and plotted. (C) LODs of BSA and EpCAM are plotted.

Supporting Figure 6

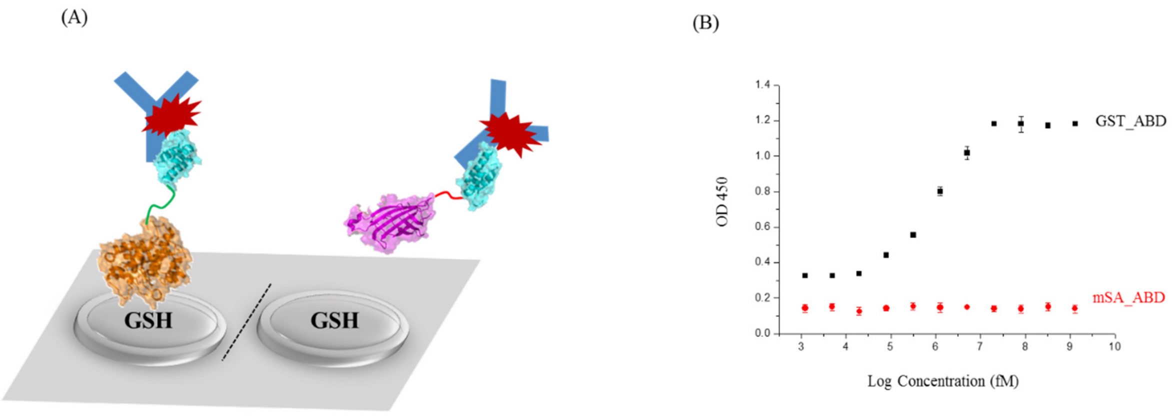

Figure S6. GST-ABD serves as an anchoring adaptor on the surface of GSH-coated plates for immobilizing antigen-capturing antibodies. (A) Scheme showing the applications of GST-ABD (left) or mSA-ABD (right) as anchoring adaptors for immobilizing antigen-capturing antibodies. GST-ABDs or mSA-ABDs are spread on the surface of GSH-coated plates, washed extensively, and saturated with the HRP-conjugated rabbit antibodies. Reactions are extensively washed and OPD and H_2O_2 are subsequently added. (B) Absorbances at 450 nm of GST-ABD anchored (black squares) and mSA-ABD anchored (red circles) reactions are measured and plotted.

Supporting Figure 7

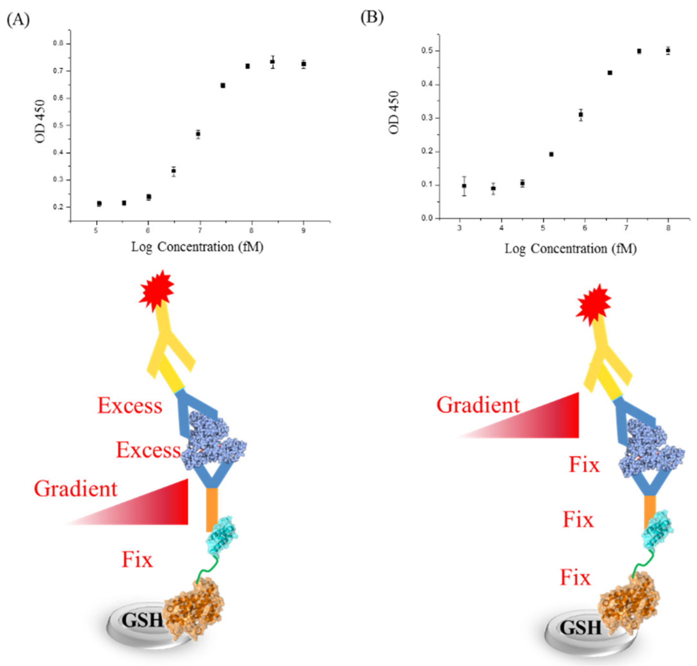

Figure S7. GST-ABD serves as an anchoring adaptor on the surface of GSH-coated plates for immobilizing anti-BSA antibodies. (A) GST-ABDs are spread on the surface of GSH-coated plates and various concentrations of anti-BSA rabbit IgGs are applied. Excess amounts of BSA, anti-BSA mouse primary IgGs, and HRP-conjugated anti-mouse secondary antibodies are subsequently added with OPD and H_2O_2. Absorbances at 450 nm are measured and plotted. (B) GST-ABDs are spread on the surface of GSH-coated plates and saturated with anti-BSA rabbit IgGs and BSA. Various amounts of anti-BSA mouse primary IgGs are added and HRP-conjugated anti-mouse secondary antibodies are subsequently added with OPD and H_2O_2. Absorbances at 450 nm are measured and plotted.

Supporting Figure 8

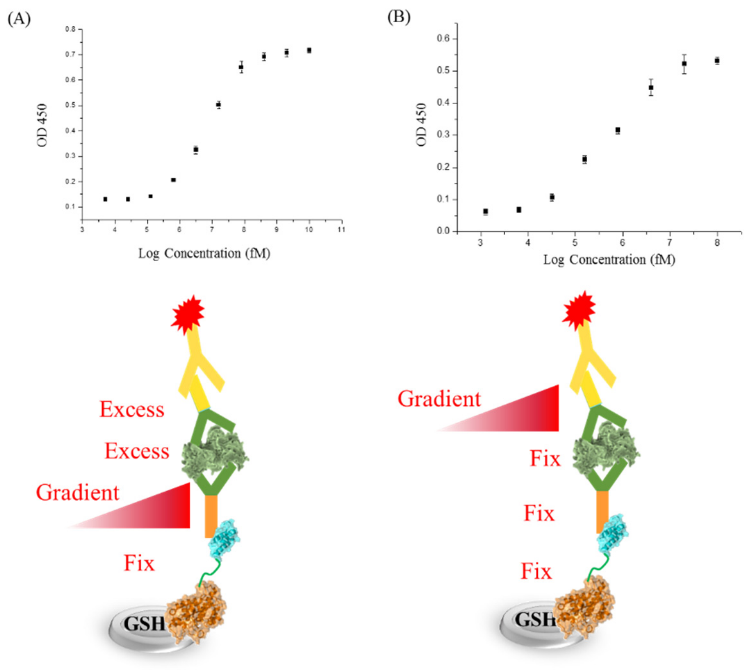

Figure S8. GST-ABD serves as an anchoring adaptor on the surface of GSH-coated plates for immobilizing anti-EpCAM antibodies. (A) GST-ABDs are spread on the surface of GSH-coated plates and various concentrations of anti-EpCAM rabbit IgGs are applied. Excess amounts of EpCAM, anti-EpCAM mouse primary IgGs, and HRP-conjugated anti-mouse secondary antibodies are subsequently added with OPD and H_2O_2. Absorbances at 450 nm are measured and plotted. (B) GST-ABDs are spread on the surface of GSH-coated plates and saturated with anti-EpCAM rabbit IgGs and EpCAM. Various amounts of anti-EpCAM mouse primary IgGs are added and HRP-conjugated anti-mouse secondary antibodies are subsequently added with OPD and H_2O_2. Absorbances at 450 nm are measured and plotted.

Supporting Figure 9

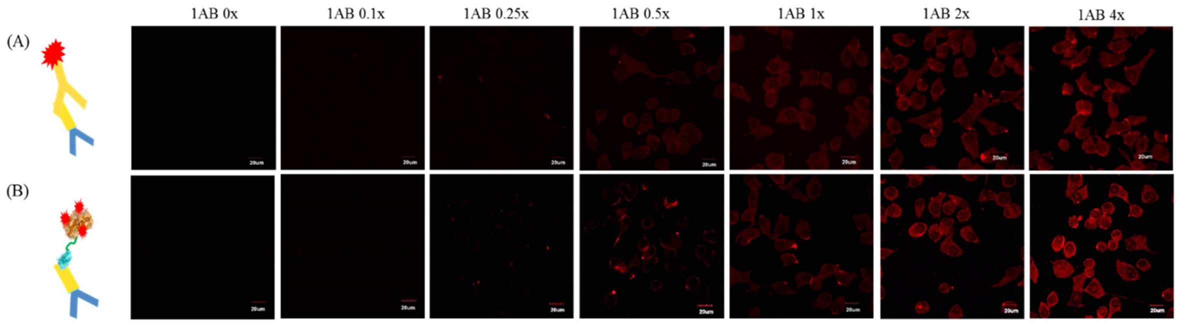

Figure S9. Tyramide signal amplification (TSA) assays with HRP-GST-ABD or general HRP-conjugated anti-mouse secondary antibodies in the presence of primary antibodies derived from mouse. KB cells overexpress integrin $\alpha\beta\gamma_3$ receptors on their surfaces. KB cells were treated with anti-integrin $\alpha\beta\gamma_3$ mouse primary IgGs and subsequently incubated with HRP-conjugated anti-mouse secondary antibodies (A) or HRP-GST-ABD (B) in the presence of TSA reagents. Treatment amounts for primary anti-integrin $\alpha\beta\gamma_3$ mouse antibodies are indicated at the top of each column. 1AB 1× represents the typical amount of primary antibodies used for TSA assays.

Supporting Figure 10

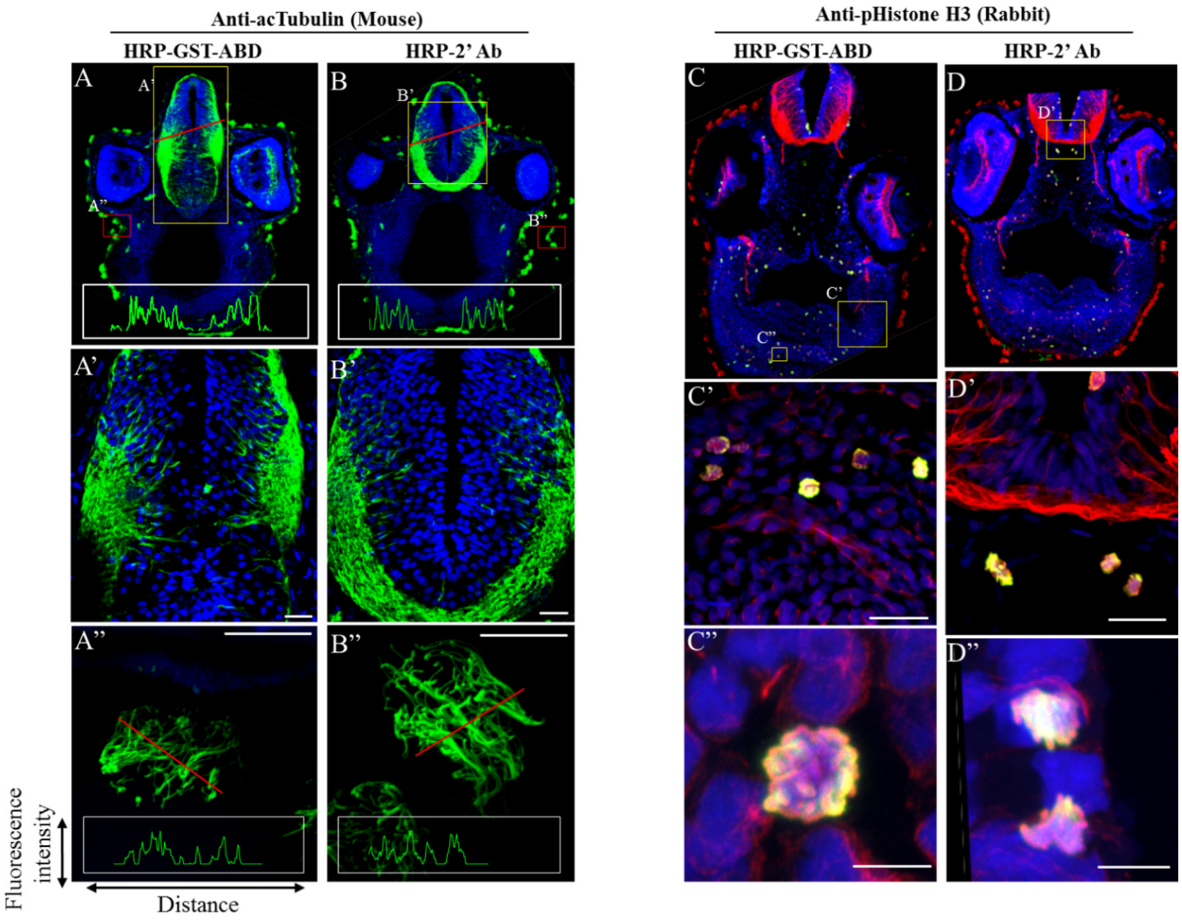

Figure S10. TSA-based immunostaining with HRP-GST-ABD results in highly specific signal amplification better than or comparable to that with classic HRP-conjugated secondary antibody. The sectioned forebrain regions are treated with complexes of the anti-acetylated tubulin primary antibodies derived from mouse and either HRP-GST-ABDs (A) or HRP-conjugated secondary antibodies (B) and then the nerve fibers are detected with a TSA assay. The nerve fibers in the forebrain are clearly stained green in both HRP-GST-ABD-treated (A') and HRP-conjugated secondary antibody-treated (B') samples. The ciliary axonemes are also specifically stained green in multiciliated cells (A" and B"). The proliferating cells are detected with complexes of anti-phospho-histone H3 primary antibodies derived from rabbits and either the HRP-GST-ABD (C) or HRP-conjugated secondary antibodies (D) by TSA-based staining. The scale bars in A'- D' and A"- D" are 20 μm and 5 μm, respectively. The insets in (A, A", B and B") show the intensity plots of the indicated regions marked by red lines.

Supporting Figure 11

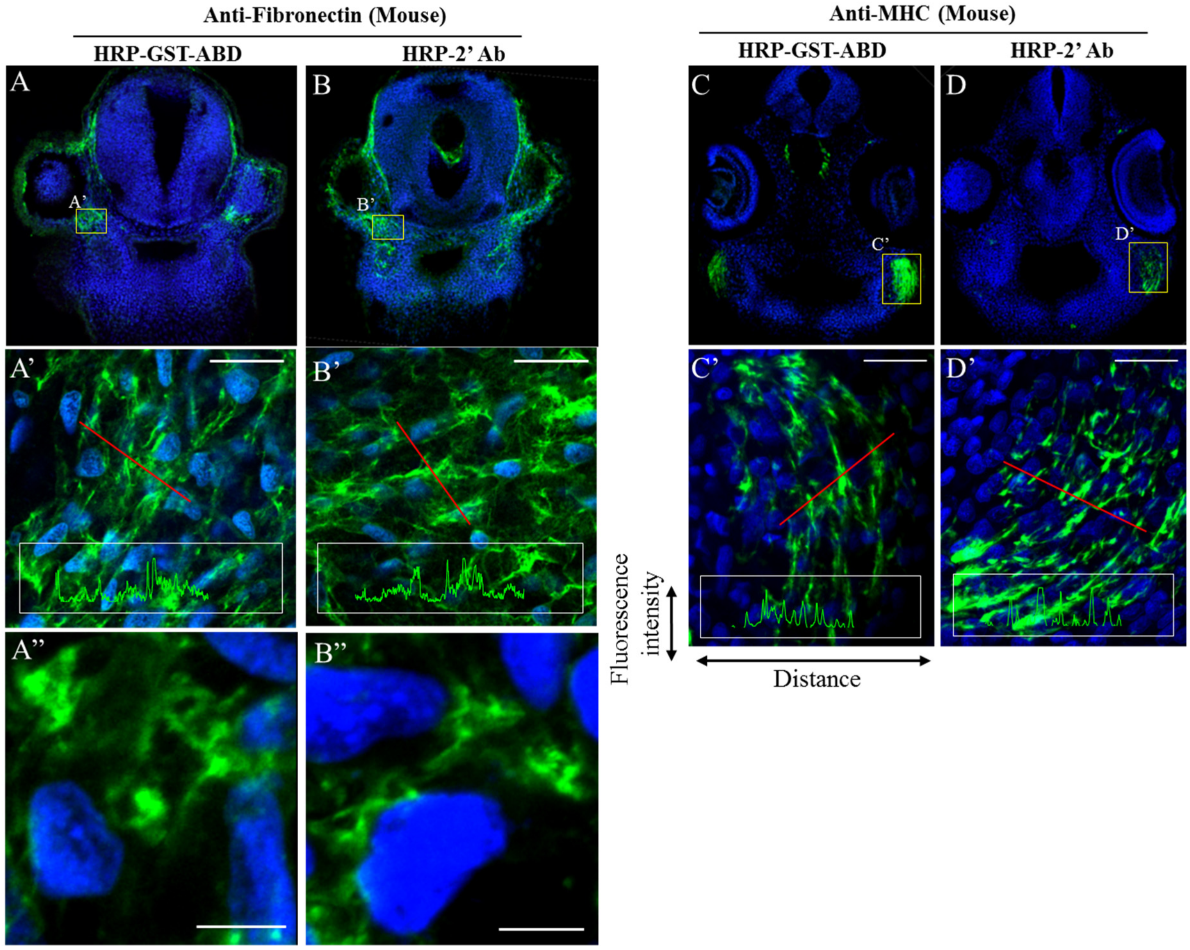

Figure S11. TSA-based immunostaining with HRP-GST-ABD resulted in highly specific signal amplification comparable to that with classic HRP-conjugated secondary antibody. The extracellular matrices (A-B) and muscles (C-D) were visualized with either HRP-GST-ABD (A, C) or HRP-secondary antibody (B, D) by using an anti-fibronectin antibody (A, B) or anti-myosin heavy chain (MHC) antibody (C, D), respectively, as mouse primary antibodies. The scale bars in A'- D' and A" - B" are 20 µm and 5 µm, respectively. The insets in (A, A", B, B", C and C") show the intensity plots of the indicated regions marked by red lines.

Supporting Figure 12

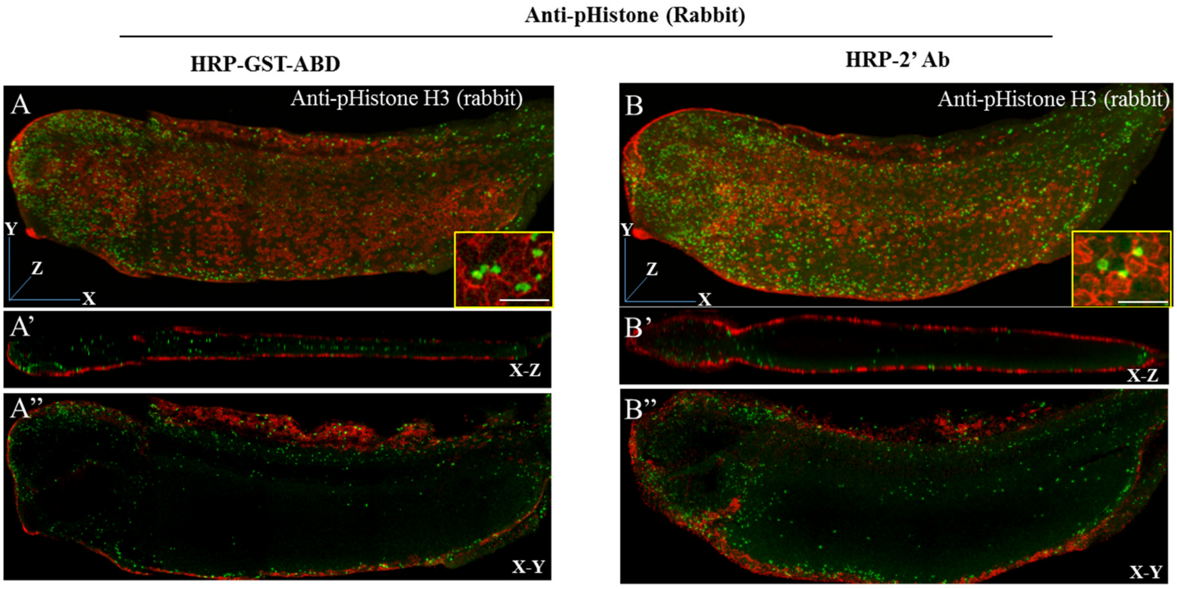

Figure S12. TSA-based immunostaining with HRP-GST-ABD result in highly specific signal amplification comparable to that with classic HRP-conjugated secondary antibody. The anti-phospho-histone H3 antibody is used to stain whole mount embryos green. HRP-GST-ABD successfully stained the whole mount samples (A) compared to the HRP-conjugated secondary antibody (B). The confocal sections show that HRP-GST-ABD penetrates the tissues efficiently and stains the internal targets specifically (A', A"). An anti-actin antibody is used to stain the cell boundaries red. The scale bars in the insets are 20 μm.
